# Optimization of Medication Regimens in Patients with Type 2 Diabetes and Clinical Atherosclerotic Cardiovascular Disease

**DOI:** 10.3390/pharmacy9040186

**Published:** 2021-11-17

**Authors:** Jarred Prudencio, Paige Cajudoy, Donald Waddell

**Affiliations:** 1 Department of Pharmacy Practice, The Daniel K. Inouye College of Pharmacy, University of Hawaii at Hilo, Hilo, HI 96720, USA; 2 The Daniel K. Inouye College of Pharmacy, University of Hawaii at Hilo, Hilo, HI 96720, USA; paigesc@hawaii.edu (P.C.); waddell2@hawaii.edu (D.W.)

**Keywords:** diabetes, cardiovascular disease, clinical pharmacist, rural, medication optimization, comprehensive medication management

## Abstract

The American Diabetes Association recommends that patients with type II diabetes and atherosclerotic cardiovascular disease be prescribed an SGLT-2 inhibitor or GLP-1 agonist for cardioprotective benefit. This project assessed the use of these medications in this patient population in a rural clinic by measuring prescribing rates of SGLT-2/GLP-1 therapy before and after pharmacist interventions. Of the 60 patients identified at baseline, 39.39% (13/33) managed by a pharmacist were prescribed SGLT-2/GLP-1 therapy compared to the 14.81% (4/27) who had not seen a pharmacist (*p* = 0.025). Of the 43 patients that were not on SGLT-2/GLP-1 therapy at baseline, 13 were lost to follow-up and 13 had contraindications. For the 17 remaining patients, pharmacists recommended initiating SGLT-2/GLP-1 therapy and were able to successfully initiate therapy for 9 patients (52.94%). Pharmacist interventions improved the prescription rates from a baseline of 36.17% (17/47) to 55.3% (26/47) (*p* = 0.002), with SGLT-2/GLP-1 therapy contraindicated in 27.66% (13/47) of patients. This suggests that patients managed by a pharmacist have medication regimens that were optimized at a greater rate and pharmacists can have a positive impact on the appropriate medication usage in this population.

## 1. Introduction

Diabetes mellitus is a chronic condition characterized by persistently elevated blood glucose levels [1]. Type II diabetes mellitus, which accounts for 90–95% of all diabetes cases, is distinguished by insulin resistance [2,3]. Initially, insulin production is increased to compensate for the insulin resistance, then insulin production may gradually decrease and produce chronic hyperglycemia [3].

Chronic hyperglycemia in type II diabetes can result in microvascular and macrovascular complications. Microvascular complications include diabetic retinopathy, nephropathy and neuropathy, while macrovascular complications include coronary artery disease, peripheral artery disease, myocardial infarction, and stroke [4]. Macrovascular complications are a grave concern, as the leading cause of morbidity and mortality in those with type II diabetes is atherosclerotic cardiovascular disease (ASCVD) [1]. ASCVD, which is defined as coronary heart disease (CHD), cerebrovascular disease or peripheral artery disease of atherosclerotic origin, is associated with type II diabetes, although the exact pathophysiology of how the condition increases the possibility of atherosclerosis is not known [1,4]. Additionally, type II diabetes commonly occurs in individuals with metabolic syndrome, which is a combination of abdominal obesity, hypertension, hyperlipidemia, and increased coagulability, which can also increase the risk of ASCVD [4].

According to a meta-analysis, diabetes doubles the risk of CHD, stroke, and deaths caused by other vascular diseases [5,6]. With the high prevalence of ASCVD in diabetes, the prevention and management of ASCVD is crucial in reducing the morbidity and mortality of patients with type II diabetes. Sodium–glucose cotransporter-2 (SGLT-2) inhibitors and glucagon-like peptide-1 receptor (GLP-1) agonists are classes of antidiabetic agents that have demonstrated cardiovascular benefits [7]. SGLT-2 inhibitors prevent glucose reabsorption in the kidneys and cause glucose to be excreted in the urine [8]. GLP-1 is an incretin, a hormone produced by the intestinal mucosa, which increases the secretion of insulin and lowers blood glucose levels after oral glucose consumption [9,10]. GLP-1 agonists cause increased insulin secretion, slowing gastric emptying and suppressing glucagon secretion [9,10].

In addition to their glycemic-lowering properties, SGLT-2 inhibitors and GLP-1 agonists have been found to have cardiovascular benefits in patients with type II diabetes. In 2008, the Food and Drug Administration (FDA) issued a Guidance for Industry, requiring that drug developers assess antidiabetic medication for its impact on cardiovascular risk via cardiovascular outcome trials (CVOTs) [11]. There have been multiple randomized controlled trials that have assessed the cardiovascular impact of GLP-1 agonists compared to placebo [12,13,14,15,16,17]. SGLT-2 inhibitor CVOTs were also conducted to assess cardiovascular risk compared to placebo [18,19,20]. The use of GLP-1 agonists and SGLT-2 inhibitors in patients with type II diabetes and established ASCVD was first recommended in the “Standards of Medical Care in Diabetes 2017” by the American Diabetes Association (ADA) [21]. The recommendation was the addition of either empagliflozin or liraglutide to metformin in patients with “long-standing suboptimally controlled” type II diabetes and established ASCVD, as these were the first CVOTs published that demonstrated cardiovascular benefits [21]. However, this was a lower recommendation, and use was primarily centered around A1c lowering. With the publication of ADA “Standards of Medical Care in Diabetes 2019”, semaglutide, exenatide, and canagliflozin were added as additional agents that could be used in combination with metformin in patients with established ASCVD [22]. At present, the ADA “Standards of Medical Care in Diabetes 2021” continues to recommend that patients with both type II diabetes and ASCVD be prescribed either an SGLT-2 inhibitor or a GLP-1 agonist for cardioprotective benefit, in addition to metformin, regardless of A1c [7].

Given that the evidence and strength of recommendations for the use of SGLT-2 inhibitors or GLP-1 agonists in patients with type II diabetes and clinical ASCVD has only increased in recent years, it is possible that clinicians may experience clinical inertia when prescribing these medications for cardiovascular risk reduction. This project was conducted in a rural primary care clinic, with the aim of assessing the baseline use of these medications in patients with type II diabetes and ASCVD, and the impact that clinical pharmacists can have on therapeutic medication optimization. The study also aimed to improve the prescription rates of these medications via clinical pharmacist interventions.

The study occurred in the East Hawaii Health Clinic, a rural primary care clinic, where clinical pharmacists work as part of the interdisciplinary team. In the East Hawaii Health Clinic, clinical pharmacists provided comprehensive medication management (CMM) for patients referred by their primary care providers. This model of CMM service has been shown to have a positive impact on chronic disease state management [23,24,25]. CMM involves the performance of medication reconciliations, assessing the safety and efficacy of patient’s therapeutic regimens, and making adjustments to regimens as necessary to optimize the medication regimens. Clinical pharmacists leverage a collaborative practice agreement with the faculty physicians of the clinic to efficiently adjust medication regimens. Clinical pharmacists also provide patient education on how to manage chronic conditions through lifestyle and pharmacologic interventions. In addition to CMM, clinical pharmacists further contribute to the interdisciplinary team by educating family medicine residents and faculty on current treatment recommendations for chronic conditions, updates in relevant clinical practice evidence, and overall pharmacotherapy.

## 2. Materials and Methods

There were two phases to this study. First, patients with type II diabetes and clinical ASCVD were identified via electronic medical record data at baseline. Baseline data on these patients were gathered, and the prescription rates of SGLT-2 inhibitors and GLP-1 agonists were assessed in this population. The first phase of the project was conducted from February to July 2020. Secondly, patients without SGLT-2 inhibitors or GLP-1 agonists were assessed by the clinical pharmacists, and recommendations were made to initiate SGLT-2 inhibitors or GLP-1 agonists when appropriate. The second phase of the project was conducted from July 2020 to June 2021. Prescription rates of SGLT-2 inhibitors and GLP-1 agonists were then reassessed in this study population. 

Patients were included in the study at baseline if they were patients of the East Hawaii Health Clinic and had a documented diagnosis of both type II diabetes and a confirmed clinical atherosclerotic event, including coronary artery disease, peripheral artery disease, myocardial infarction, or stroke. No additional exclusion criteria were set for this study. 

The primary objective of this project was to evaluate the pharmacist’s role in therapy optimization for patients with type II diabetes and clinical ASCVD in a rural primary care clinic, measured by baseline and post-intervention prescription rates of SGLT-2 inhibitors and GLP-1 agonists. Baseline rates were assessed by determining the number of patients that were prescribed an SGLT-2 inhibitor of GLP-1 agonist therapy at the start of the study analysis. Patients were categorized into two groups: those who had at least one CMM appointment with a clinical pharmacist and those who were not referred for CMM. The baseline prescribing rates of SGLT-2/GLP-1 therapy were assessed between these two groups.

After baseline analysis, clinical pharmacists reviewed medical records for the remainder of patients who were not prescribed an SGLT-2 inhibitor or GLP-1 agonist at baseline and made recommendations to initiate an SGLT-2 inhibitor or GLP-1 agonist when appropriate. To determine if SGLT-2 or GLP-1 agonist therapy were appropriate, clinical pharmacists reviewed factors such as the patient’s current therapeutic regimen, renal function, past medical history, and insurance coverage. Once an SGLT-2 inhibitor or GLP-1 agonist therapy was deemed to be appropriate, recommendations for therapy initiation were made at each patient’s upcoming follow-up appointment. If a patient did not have a follow-up appointment scheduled with their primary care provider or a clinical pharmacist, the patient would be contacted to schedule an appointment. The recommendations to initiate therapy were given to either the primary care provider or a clinical pharmacist, with preference given to the clinician that was scheduled to see the patient next. Recommendations were delivered through the electronic medical record prior to the patient appointment. After recommendations to initiate SGLT-2 inhibitors or GLP-1 agonists were made, and electronic medical records were monitored to determine if the recommendations were accepted by the clinician and patient after the appointment. Post-intervention prescribing rates were then assessed. Continuous variables were analyzed using the *t*-test and categorical data were examined using Fisher’s exact test. The study analysis was reviewed and deemed exempt by the University of Hawaii Institutional Review Board.

## 3. Results

**Figure 1:**
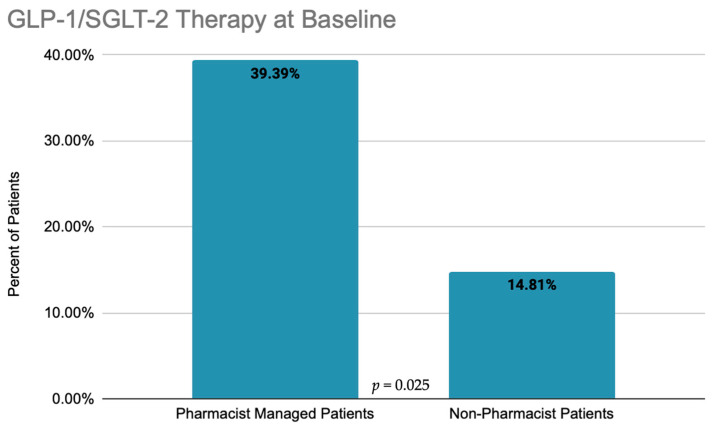
Baseline comparison of GLP-1 agonist and SGLT-2 inhibitor use in pharmacist-managed cohort vs. non-pharmacist cohort.

A total of 60 patients were identified with both type II diabetes mellitus and ASCVD, and included in the analysis. At baseline, 33 patients had been seen by a clinical pharmacist for CMM while the remaining 27 had not been seen by a clinical pharmacist. The baseline demographics of the patients are detailed in Table 1. A total of 28.3% (17/60) of patients were prescribed GLP-1 agonist/SGLT-2 inhibitor therapy at baseline. Of the patients managed by a pharmacist, 39.39% (13/33) were appropriately prescribed GLP-1 agonist/SGLT-2 inhibitor therapy. When compared to the cohort of patients who had not seen a pharmacist, 14.81% (4/27) patients had been prescribed GLP-1/SGLT-2 therapy. A comparison of the baseline prescription rates of these two cohorts is depicted in Figure 1 (*p =* 0.025).

A total of 43 patients were not prescribed a GLP-1 agonist or an SGLT-2 inhibitor at baseline. In the second phase of the study, after reviewing the patients not on a GLP-1 agonist/SGLT-2 inhibitor at baseline, 13 patients were identified as lost to follow-up as they moved to a different healthcare system. Of the remaining 30 patients, clinical pharmacists reviewed medical records and it was deemed that GLP-1 agonist/SGLT-2 inhibitor therapy was inappropriate for an additional 13 patients, either due to kidney dysfunction, cost concerns, or other reasons (27.66%). Recommendations were made to the clinicians to start the remaining 17 patients on a GLP-1 agonist/SGLT-2 inhibitor. 

**Figure 2:**
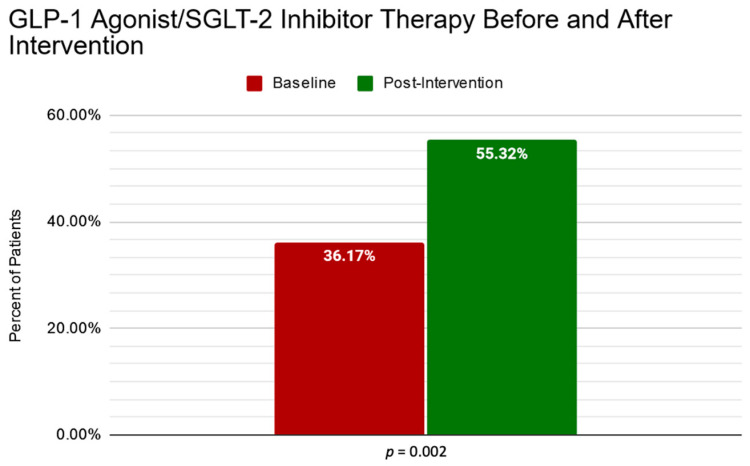
Comparison of GLP-1 agonist/SGLT-2 inhibitor prescribing rates from baseline to post-intervention.

Within 3 months after the recommendations, 9 of the 17 patients (52.94%) had been successfully started on GLP-1/SGLT-2 therapy. Post-intervention prescription rates were then assessed, leaving a total of 55.3% (26/47) of patients prescribed a GLP-1 agonist/SGLT-2 inhibitor, increased from a baseline of 36.17% (17/47) when excluding the patients that were eventually lost to follow-up (*p* = 0.002). The changes in prescription rates from baseline to post-intervention are depicted in Figure 2.

Of note, 56.67% (16/30) of these patients that were not prescribed a GLP-1 agonist/SGLT-2 inhibitor at baseline already had achieved a hemoglobin A1c within goal of <7%. Of those patients with controlled hemoglobin A1c levels, 50% (8/16) were not prescribed metformin at baseline and 31.25% (5/16) controlled their glycemic levels solely through diet and lifestyle modifications.

## 4. Discussion

The results of this study demonstrate two instances of clinical pharmacists having a positive impact on therapeutic optimization. Firstly, a higher GLP-1 agonist/SGLT-2 inhibitor prescription rate among patients managed by clinical pharmacists compared to those without a clinical pharmacist was observed at baseline. Secondly, a review of clinical pharmacists and interventions led to an improvement in the prescription rates of these medications from baseline. These results add to the growing body of evidence that supports the integration of clinical pharmacists’ into ambulatory care settings by demonstrating the additional benefits of these services.

The effect that pharmacists can have in optimizing therapeutic regimens has been investigated in numerous studies, which produced findings that are consistent with the results of this study. A systematic review and meta-analysis of the impact that pharmacists can have on diabetes care in an ambulatory care setting found that therapeutic outcomes, specifically the mean difference in hemoglobin A1c, systolic blood pressure, and low-density lipoprotein cholesterol, were significantly improved after interventions were made by pharmacists [26]. Additionally, a similar pilot study has investigated the impact of pharmacist intervention on the prescription rates of GLP-1 agonist/SGLT-2 inhibitor therapy in patients with both type II diabetes and ASCVD [27]. The pilot study investigated the impact of pharmacist intervention through the implementation of a pharmacist-designed protocol based on the ADA “Standards of Medical Care in Diabetes 2018”, the current guidelines at the time of the study [27]. Based on the recommendations at the time, empagliflozin, canagliflozin, and liraglutide were the only medications included in the study protocol. Although the pilot study, differing from this current study by investigating indirect pharmacist intervention via a pharmacist-designed prescription algorithm, both studies demonstrated a significant increase in appropriate GLP-1 agonist/SGLT-2 inhibitor therapy after intervention by a clinical pharmacist [27].

There were several limitations of this study that should be noted. The first limitation of this study is the method of data collection and review, which was conducted solely through electronic medical records. Electronic medical records may not accurately document the patients’ complete medical history and/or other factors that may affect the clinician’s decision to prescribe GLP-1 agonist/SGLT-2 inhibitor therapy, such as history of nonadherence or issues with drug costs. Without a full and complete clinical picture of each patient, it is possible that more patients had reasons to not use a GLP-1 agonist/SGLT-2 inhibitor therapy than reported in this study. Second, several post-intervention prescriptions of GLP-1 agonist/SGLT-2 inhibitor therapy relied on patient amenability to scheduling an appointment for a clinic visit. Prescriptions in this study were made during clinic visits, either with a CMM pharmacist or with a primary care provider. Patients that were determined to be eligible for GLP-1/SGLT-2 therapy but did not have an upcoming appointment scheduled were contacted by the clinic’s clerical staff. However, several patients were not interested in scheduling an appointment, and this refusal of or delay in a follow-up appointment leads to the loss of opportunity for a potential initiation of GLP-1 agonist/SGLT-2 inhibitor therapy. Additionally, patient-centered care is a practice in this clinic and, despite their education, patients may decline additional medications. The final limitation of this study is the inclusion of patients with controlled hemoglobin A1c levels, indicated by a hemoglobin A1c of <7%, as recommended by the ADA “Standards of Medical Care in Diabetes 2021” [7]. A total of 16 patients had levels within their goal, with 50% of these patients not taking metformin. For many patients, metformin was not prescribed, as their diabetes was controlled through diet and lifestyle modifications. In accordance with the ADA 2021 guidelines, GLP-1 agonist/SGLT-2 inhibitor therapy should be initiated with metformin, given that most patients in the CVOTs were on metformin therapy [7]. To initiate a GLP-1 agonist/SGLT-2 inhibitor for this group of patients, metformin initiation would also have to be considered. Initiating two antidiabetic medications in patients who have had controlled type II diabetes without the use of medication is unlikely to occur, as some patients or clinicians may perceive this as unnecessary or excessive.

It must also be acknowledged that this study’s sample size is fairly small, due to the patient population size available in this rural clinic. Although a smaller sample size is a limitation, it can also be viewed as a benefit, particularly for other small/rural practices who can view this study as replicable in their own settings. Conducting this study on a larger population would also be beneficial. Additionally, it would be of value for a future study to assess if patients on GLP-1/SGLT-2 therapy demonstrated improved clinical outcomes, as seen in the CVOTs. 

With the successful improvement in the prescription rates of appropriate medication use demonstrated in this study using electronic medical record review and pharmacist interventions, further studies in this clinic are planned, following the same methods. Pharmacists may review medical records for patients in this clinic while focusing on additional targeted medications for other conditions or situations, such as focusing on statin-prescribing rates in patients with ASCVD or diabetes, guideline-directed medication therapy for patients with heart failure with reduced ejection fraction, or the avoidance of high-risk medication in the elderly. Further efforts will be made to solidify a protocolized process for clinical pharmacists to provide a continuous improvement in the quality of the prescription of medication in this clinic.

## 5. Conclusions

The results of this study suggest that patients with type II diabetes and clinical ASCVD, who are managed by a clinical pharmacist for CMM, have had significantly greater rates of medication optimization compared to patients without a pharmacist. This adds to the evidence supporting the integration of clinical pharmacists in outpatient clinical settings to work alongside other healthcare providers in improving team-based healthcare. Additionally, it is noted that clinical pharmacists can have a positive impact on the appropriate medication usage in this patient population.

## Figures and Tables

**Table 1:** Baseline Characteristics (*n* = 60).

	Pharmacist Managed Group (*n* = 33)	Non-Pharmacist Group (*n* = 27)
Age, average (range), years	64.85 (44 to 91)	64.43 (32 to 78)
Female Gender (%)	54.55%	57.14%
A1c, average (range), %	7.72 (4.8 to 10.7)	7.21 (5.7 to 10.4)
GLP-1 Agonist use	12 (36.36%)	2 (7.4%)
SGLT-2 Inhibitor use	3 (9.09%)	3 (11.11%)

## Data Availability

The datasets generated during and/or analysed during the current study are available from the corresponding author on reasonable request.

## References

[1] Diabetes World Health Organization. https://www.who.int/health-topics/diabetes#tab=tab_1.

[2] Riddle M.C., Bakris G., Blonde L., American Diabetes Association (2021). 2. Classification and diagnosis of diabetes: Standards of medical care in diabetes-2021. Diabetes Care.

[3] Goyal R., Jialal I. (2021). Diabetes Mellitus Type 2.

[4] Fowler M.J. (2008). Microvascular and macrovascular complications of diabetes. Clin. Diabetes.

[5] Sarwar N., Gao P., Seshasai S.R., Gobin R., Kaptoge S., Di Angelantonio E., Ingelsson E., Lawlor D.A., Selvin E., Emerging Risk Factors Collaboration (2010). Diabetes mellitus, fasting blood glucose concentration, and risk of vascular disease: A collaborative meta-analysis of 102 prospective studies. Lancet.

[6] International Diabetes Federation (2019). IDF Diabetes Atlas.

[7] Riddle M.C., Bakris G., Blonde L., American Diabetes Association (2021). 9. Pharmacologic approaches to glycemic treatment: Standards of medical care in diabetes-2021. Diabetes Care.

[8] Kalra S. (2014). Sodium glucose co-transporter-2 (SGLT2) inhibitors: A review of their basic and clinical pharmacology. Diabetes Ther..

[9] Collins L., Costello R.A. (2021). Glucagon-Like Peptide-1 Receptor Agonists.

[10] Hinnen D. (2017). Glucagon-like peptide 1 receptor agonists for type 2 diabetes. Diabetes Spectr..

[11] Chong W. FDA Background Document: Endocrinologic and Metabolic Drugs Advisory Committee Meeting. https://www.fda.gov/media/121272/download.

[12] Andrikou E., Tsioufis C., Andrikou I., Leontsinis I., Tousoulis D., Papanas N. (2019). GLP-1 receptor agonists and cardiovascular outcome trials: An update. Hellenic J. Cardiol..

[13] Marso S.P., Daniels G.H., Brown-Frandsen K., Kristensen P., Mann J.F.E., Nauck M.A., Nissen S.E., Pocock S., Poulter N.R., Ravn L.S. (2016). Liraglutide and cardiovascular outcomes in type 2 diabetes. N. Eng. J. Med..

[14] Marso S.P., Bain S.C., Consoli A., Eliaschewitz F.G., Jódar E., Leiter L.A., Lingvay I., Rosenstock J., Seufert J., Warren M.L. (2016). Semaglutide and cardiovascular outcomes in patients with type 2 diabetes. N. Eng. J. Med..

[15] Gerstein H.C., Colhoun H.M., Dagenais G.R., Diaz R., Lakshmanan M., Pais P., Probstfield J., Riesmeyer J.S., Riddle M.C., Rydén L. (2019). Dulaglutide and cardiovascular outcomes in type 2 diabetes (REWIND): A double-blind, randomised placebo-controlled trial. Lancet.

[16] Holman R.R., Bethel M.A., Mentz R.J., Thompson V.P., Lokhnygina Y., Buse J.B., Chan J.C., Choi J., Gustavson S.M., Iqbal N. (2017). Effects of once-weekly exenatide on cardiovascular outcomes in type 2 diabetes. N. Eng. J. Med..

[17] Pfeffer M.A., Claggett B., Diaz R., Dickstein K., Gerstein H.C., Køber L.V., Lawson F.C., Ping L., Wei X., Lewis E.F. (2015). Lixisenatide in patients with type 2 diabetes and acute coronary syndrome. N. Eng. J. Med..

[18] Zinman B., Wanner C., Lachin J.M., Fitchett D., Bluhmki E., Hantel S., Mattheus M., Devins T., Johansen O.E., Woerle H.J. (2015). Empagliflozin, cardiovascular outcomes, and mortality in type 2 diabetes. N. Eng. J Med..

[19] Neal B., Perkovic V., Mahaffey K.W., de Zeeuw D., Fulcher G., Erondu N., Shaw W., Law G., Desai M., Matthews D.R. (2017). Canagliflozin and cardiovascular and renal events in type 2 diabetes. N. Eng. J. Med..

[20] Wiviott S.D., Raz I., Bonaca M.P., Mosenzon O., Kato E.T., Cahn A., Silverman M.G., Zelniker T.A., Kuder J.F., Murphy S.A. (2019). Dapagliflozin and cardiovascular outcomes in type 2 diabetes. N. Eng. J. Med..

[21] American Diabetes Association (2017). 8. Pharmacologic approaches to glycemic treatment: Standards of medical care in diabetes-2017. Diabetes Care.

[22] American Diabetes Association (2019). 9. Pharmacologic approaches to glycemic treatment: Standards of medical care in diabetes-2019. Diabetes Care.

[23] Prudencio J., Kim M. (2020). Diabetes-related patient outcomes through comprehensive medication management delivered by clinical pharmacists in a rural family medicine clinic. Pharmacy.

[24] Prudencio J., Cutler T., Roberts S., Marin S., Wilson M. (2018). The effect of clinical pharmacist-led comprehensive medication management on chronic disease state goal attainment in a patient-centered medical home. J. Manag. Care Spec. Pharm..

[25] Fink R.M., Mooney E.M., Saseen J.J., Billups S.J. (2019). A comparison of clinical pharmacist management of type 2 diabetes versus usual care in a federally qualified health center. Pharm. Pract..

[26] Fazel M.T., Bagalagel A., Lee J.K., Martin J.R., Slack M.K. (2017). Impact of diabetes care by pharmacists as part of health care team in ambulatory settings: A systematic review and meta-analysis. Ann. Pharmacother..

[27] Freudenberg D.L., Covington L.P., Young R.B., Lopez N.D., Patel M.V., MacLaughlin E.J. (2020). Impact of a pharmacist-driven protocol to improve guideline-concordant prescribing of diabetes medications in patients with atherosclerotic cardiovascular disease: A pilot study. J. Pharm. Pract..

